# Structural and Electrotransport Properties of Perfluorinated Sulfocationic Membranes Modified by Silica and Zirconium Hydrophosphate

**DOI:** 10.3390/membranes12100979

**Published:** 2022-10-08

**Authors:** Svetlana A. Shkirskaya, Natalia A. Kononenko, Sergej V. Timofeev

**Affiliations:** 1 Physical Chemistry Department, Kuban State University, 149 Stavropolskaya Str., 350040 Krasnodar, Russia; 2 JSC Plastpolymer, 32 Polyustrovskiy prospect, 195197 St. Petersburg, Russia

**Keywords:** perfluorinated sulfocationic membranes, silica, zirconium hydrophosphate, modification, conductivity, electroosmotic permeability, diffusion permeability, porosimetric curve

## Abstract

A correlation between changes in structural and electrotransport properties of membranes after modification by silica and zirconium hydrophosphate was established. The total water volume, volume fraction of the free water in the membrane and the volume fraction of the water having high binding energy were considered as structural characteristics, which were found from the curves of water distribution on the water binding energy and the effective pore radii. The conductivity, diffusion and electroosmotic permeabilities were investigated as electrotransport properties. The influence of the modifier type on the current flow paths in the membrane was analyzed within the framework of the extended three-wire model. It has been established that the treatment of membranes with alcohol before the intercalation of a modifier leads to the appearance of cavities with an effective size of more than 100 nm filled with free water with the binding energy less than 10 J/mol. It is accompanied with an increase in the diffusion permeability of hybrid membranes by approximately 3–6 times in NaCl and HCl solutions, which limits the application of such materials in proton exchange membrane fuel cells. The different conditions of modification of perfluorinated membranes with similar properties by the dopant with same type allow for the preparation of the hybrid materials for various applications such as electrodialysis concentration or electric current generation devices.

## 1. Introduction

Bulk or surface modification of commercial membranes is an effective approach to obtain materials with required properties for different applications [1,2,3,4,5,6,7,8]. The most prospective direction of improving performance of ion-exchange membranes in membrane devices is their modification by nanoparticles of organic and inorganic compounds resulting in enhancement of proton conductivity and water uptake of membranes at low relative humidity [9,10,11,12,13,14,15,16]. Modification of perfluorinated membrane by nanoparticles can be useful not only for improving the fuel cells performance, but also for separation processes if those processes are carried out at high temperatures and in acidic environments [17,18,19]. Methods of nanoparticles intercalation into the perfluorinated membrane matrix may dramatically affect both its structural characteristics and transport properties. 

The modifying conditions have the same strong influence on the membrane properties as well as the modifier nature. The most commonly used dopants are hydrated oxides of polyvalent elements (silicon, zirconium, etc.) [20]. The hydrophilic surface of dopants can increase in the water uptake of membranes and enhance the proton conductivity even at low humidity. There are two principal ways to incorporate nanoparticles of various inorganic materials into the membrane structure. The first one involves the synthesis of nanoparticles followed by membrane casting from mixed solution of polymer and dopant. Another method is the synthesis of inorganic particles directly (in situ) in the membrane matrix where the membrane pores are used as nanoreactors. In this case, the membrane structure can be pre-expanded by treatment with alcohol to increase the amount of dopant. However, such pretreatment of the membrane during modification can significantly affect the transport properties of the hybrid membrane.

It is known that properties of hybrid membranes on the basis of perfluorinated matrix and silica or zirconium hydrophosphate (HZP) essentially depend on the precursor concentration, temperature, precipitation pH and ultrasonic treatment [8,21,22,23,24,25,26,27,28,29,30,31,32,33]. Membranes with high water uptake and improved proton conductivity are usually prepared. However, under appropriate conditions, where the membrane is exposed to high temperature, it is possible to obtain an organic-inorganic hybrid membrane with reduced water content and low electroosmotic permeability [21]. While the membranes with enhanced water uptake are interesting for fuel cells applications, the membranes with reduced water uptake are promising for the use in membrane electrolysis and electrodialysis [34]. 

The purpose of this work was to find relations between structural characteristics and transport properties of perfluorinated membranes modified by HZP and silica under different conditions and reveal the role of pretreatment with alcohol before modification and heating after the synthesis of silica nanoparticles in the membrane matrix in membrane characteristics.

## 2. Materials and Methods

### 2.1. Materials

The objects of research were perfluorinated sulfonated cation-exchange Nafion 115 (Dupont, Wilmington, DE, USA) and MF-4SK (JSC Plastpolymer, St. Petersburg, Russia) membranes having similar physicochemical characteristics (Table 1). Silica and zirconium hydrophosphate were membrane modifiers. The HZP-modified membrane was prepared based on MF-4SK membrane. Tetraethoxysilane Si(C_2_H_5_O)_4_ and zirconium oxychloride ZrOCl_2_·8H_2_O were used as precursor to prepare SiO_2_ and Zr(HPO_4_)_2_ into membrane. Solutions of hydrochloric acid, ethanol and ammonia solution (KhimMed, Russia, extra-pure grade) were used in this work. Deionized water was used for membranes synthesis and washing.

### 2.2. Membrane Modification

Hybrid membranes were prepared by the in situ introduction of a dopant into the membrane perfluorinated matrix [33]. Pore walls of membranes can effectively sorb initial reagents and limit the reaction volume. Since the typical nanopore size is no greater than 5 nm, the formed particles are from 2 to 5 nm in diameter [2]. Additionally, pore walls isolate the resulting particles from each other and reduce surface tension forces, providing the thermodynamic stability of the nanoparticles. A Nafion membrane was used for the preparation of Nafion/SiO_2_ samples with high water uptake while MF-4SK membrane was used for the preparation of MF-4SK/SiO_2_ samples with low water uptake. 

The Nafion membrane was exposed to a solution of tetraethoxysilane in ethanol (1:4) for 3 h at room temperature under continuous stirring. Then the sample was placed in a 12 wt.% of ammonia solution for 30 min to transform the precursor into hydrated oxide. Prepared membranes were treated by 10% hydrochloric acid for 3 h at room temperature in order to provide the protonic form and washed with deionized water.

The MF-4SK membrane was pre-treated by soaking in ethanol for 24 h to extend its matrix before silica modification. The same procedures as with the Nafion were carried out for MF-4SK membrane. Then the MF-4SK membrane was kept at 100 °C to prepare a sample with reduced water content [21].

The HZP-modified hybrid membranes were manufactured in the similar way as Nafion/SiO_2_ samples. The MF-4SK membrane in H^+^-form was exposed to water-ethanol solution, then to ZrOCl_2_·8H_2_O solution; afterwards, to phosphoric acid solution to precipitate zirconium hydrophosphate Zr(HPO_4_)_2_.

### 2.3. Research Methods

Physicochemical characteristics of the initial and modified membranes were determined using standard methods described in [35]. The ion-exchange capacity (*Q*, mmol/g_dry_) was determined for H^+^-form samples of the perfluorinated membrane by titration of the H^+^-ions produced in the course of base neutralization. 

The method of standard contact porosimetry [36,37], which had been acknowledged by the International Union of Pure and Applied Chemistry [38], was applied to investigate the water volume distribution in the membrane on the water binding energy or the effective pore radii (*r*). 

The binding energy between water and membrane material (*A*, J/mol ) correlates with *r* according to formula

(1) $$A=2\upsilon_{w}\sigma \frac{\cos\theta }{r}$$

where *υ_w_* was the molar water volume, *σ* was the interfacial surface tension, *θ* was the contact (wetting) angle.

**Figure 1:**
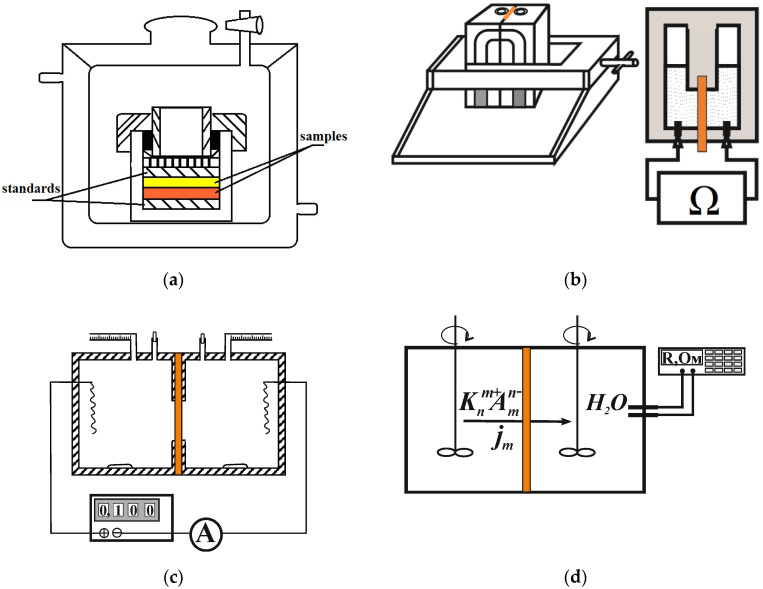
Schematic drawings of the experimental cells to determine porosimetric curve (**a**), conductivity (**b**), electroosmotic (**c**) and diffusion (**d**) permeabilities of the membrane.

The membrane samples were piled in a special clamping device (Figure 1a between two standards, having the known pore-size distribution obtained by an independent method (e.g., mercury porosimetry). After partial evaporation of water and achieving capillary equilibrium, the standards and samples were weighed, and their water volume was calculated using the mass balance. The pore radius corresponding to a given water volume was found with the use of the standards.

The maximum porosity value was determined from the obtained porosimetric curves as water volume per gram of the dry sample (*V*_0_, cm^3^/g). 

The number of H_2_O molecules per functional –SO_3_^−^ groups (*n_m_*) was calculated from the values of the *V*_0_ and exchange capacity using the equation,

(2) $$n_{m}=\frac{V_{0}}{18Q}$$

The specific internal surface area (*S*_1_, m^2^/g) was calculated by the formula [39],

(3) $$S_{1}=2\int_{r\min}^{r\max}\frac{1}{r^{2}}\left(\frac{dV}{d\ln r}\right)dr=2\int_{r\min}^{r\max}\frac{dV}{r}$$

where *r*_min_ and *r*_max_ were the minimum and maximum pore radii, respectively. 

The lower limit of integration is estimated as 1 nm, which corresponds to the lowest limit of this method applicability. The area of internal specific micropore surface (*S*_2_) with a radius less than 1 nm was approximately evaluated by the formula,

(4) $$S_{2}=\frac{2V_{\min}}{r_{\min}}$$

where *r*_min_ = 1 nm, *V*_min_ was the volume of pore with a radius less than 1 nm.

The total area of the internal specific surface (S) consists of the above-mentioned two parts: *S* = *S*_1_ + *S*_2_.

**Figure 2:**
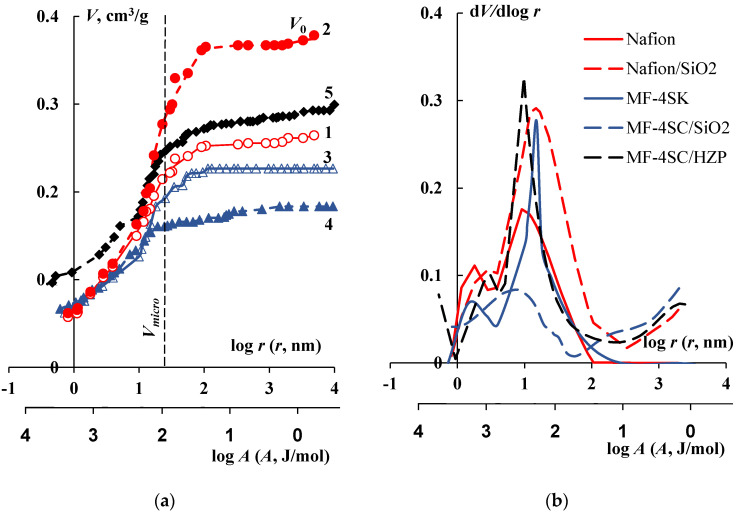
Integral (**a**) and differential (**b**) functions of water distribution on the water binding energy and the effective pore radii for the initial (1, 3) and modified (2, 4, 5) membranes: 1—Nafion; 2—Nafion/SiO_2_; 3—MF-4SK; 4—MF-4SK/SiO_2_; 5—MF-4SK/HZP.

The volume of hydrophilic unselective macropores filled with unbound, "free" water (*V_macro_*) was found from the porosimetric curve. The volume of micro and mesopores (*V_micro_*) having diameter below 50 nm [40] and nearly ideal selectivity was found as well (Figure 2). The volume fraction of macropores in swollen membrane ($\frac{V_{macro}}{V_{sw.m}}$) characterizes the membrane heterogeneity [41], and the fraction of micro and mesopores volume in the total volume of water in the membrane ($\frac{V_{micro}}{V_{0}}$) indicates the membrane ion selectivity [39].

An average distance *L* between fixed groups at interfaces was calculated using *S* value and membrane exchange capacity *Q* by the following formula,

(5) $$L=\sqrt{\frac{S}{QN_{A}}}$$

where *N_A_* was the Avogadro’s number.

The membrane conductivity (*κ_m_*, S/m) was determined from the value of membrane resistance measured as active portion of the membrane impedance with the use of the mercury-contact technique (Figure 1b).

The water transport number (*t_w_*, mol H_2_O/F) was calculated from electroosmotic permeability (*D_w_*, cm^3^/A s) of the membranes in NaCl solutions measured by the volumetric method in a two-chamber cell with reversible silver-silver chloride electrodes (Figure 1c). The water transport number was defined as the number of moles of water transported through the membrane per 1 Faraday of passed electricity and calculated by the formula,

(6) $$t_{w}=\frac{D_{w}F}{\upsilon_{w}}=\frac{\upsilon F}{S_{m}i\tau \upsilon_{w}}$$

where *υ* is the changes of water volume in the capillary tubes (cm^3^), *F* is the Faraday constant, *S_m_* is the membrane surface area (cm^2^), *i* is the current density (A/cm^2^), *τ* is the time period of the process (s), *υ_w_* is the molar water volume (18 cm^3^/mol). 

The diffusion permeability (*P*_m_, m^2^/s) was measured in two-compartment cell by diffusion of electrolyte solution through the membrane to distilled water (Figure 1d). The change in solution resistance in the chamber filled with distilled water was used to control the kinetic increase of solution concentration [35]. 

The experiments were carried out at a temperature of 25 °C and the measurement errors for all experimental characteristics amounted to 3–5%.

## 3. Results and Discussion

### 3.1. Structural Characteristics

The integral and differential curves of water volume distribution on the water binding energy and the effective pore radii for initial and modified membranes are shown in Figure 2. Some physicochemical and structural characteristics of the initial and modified membranes found from porosimetric curves are presented in Table 1. Analysis of the obtained data shows that the initial Nafion and MF-4SK membranes have similar physicochemical and structural characteristics. Both Nafion and MF-4SK membranes do not have cavities with an effective size of more than 100 nm in their structure. However, pretreatment with alcohol before modifying of the membranes leads to the appearance of cavities filled with free water with A < 10 J/mol.

The modification of perfluorinated membranes by silica under different conditions leads to the preparation of hybrid membranes with various structure. The synthesis of silica in the matrix of perfluorinated membranes results in a decrease of the membrane ion-exchange capacity but other membrane characteristics can both increase and decrease (Table 1). The Nafion membrane modification by silica causes the increase in the total volume of water (*V*_0_) by 40%, the area of specific internal surface (*S*) by 10% and the volume of pores in the range of 10–100 nm. The volume fraction of macropores ($\frac{V_{macro}}{V_{sw.m}}$) is higher in Nafion/SiO_2_ membrane than in Nafion one. Thermal treatment of hybrid MF-4SK/SiO_2_ membrane results in the decrease of the *V*_0_ value by 20%, the *n_m_* value by 15% and pores volume in the range of 10–100 nm (Figure 2b) in comparison with the initial membrane.

Preparation of the MF-4SK/HZP membrane also started with exposure to water-ethanol solution, however heat treatment was not carried out. Therefore, the resulting hybrid membrane structure is close to Nafion/SiO_2_ membrane. As can be seen from the Figure 2 and Table 1, the *V*_0_ value in hybrid membrane is 30% higher than in initial MF-4SK one due to an increase in the fraction of macropores $\frac{V_{\mathrm{macro}}}{V_{\mathrm{sw}.m}}$ in the swollen membrane. The peculiarities in the change in structural characteristics of MF-4SK/HZP membrane are due to the fact that HZP is a mineral ion-exchanger. Intercalation of the ion-exchange groups during modification leads to an increase in the *Q* value of the hybrid membrane by more than 2 times. As a result, the volume of high-energy water in the range of r ≈ 1 nm also increases. This water is included in the hydration shell of phosphoric acid groups. Despite an increase in the total water content *V*_0_, the specific water content *n*_m_ decreases by almost 2 times because of the *Q* value growth. The presence of an additional number of ion-exchange groups in the membrane leads to a decrease in the *L* value by 25% despite an increase in the *S* value from 194 to 250 m^2^/g. At the same time, the *S* and the *L* values change insignificantly for other hybrid membranes (Table 1). High values of the $\frac{V_{micro}}{V_{0}}$ parameter allow for the assumption that all modified membranes keep the ion selectivity close to the initial membranes.

### 3.2. Transport Properties

**Figure 3:**
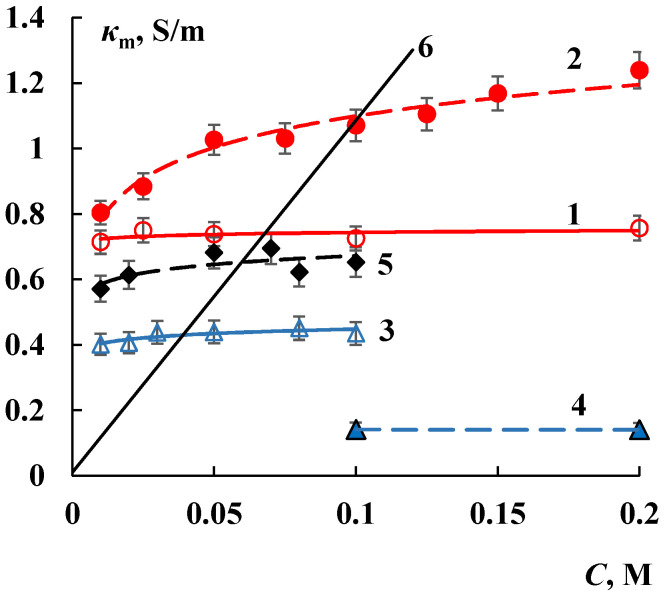
Concentration dependences of membrane conductivity in NaCl solutions: 1—Nafion, 2—Nafion/SiO_2_, 3—MF-4SK, 4—MF-4SK/SiO_2_, 5—MF-4SK/HZP, 6—NaCl solution.

**Figure 4:**
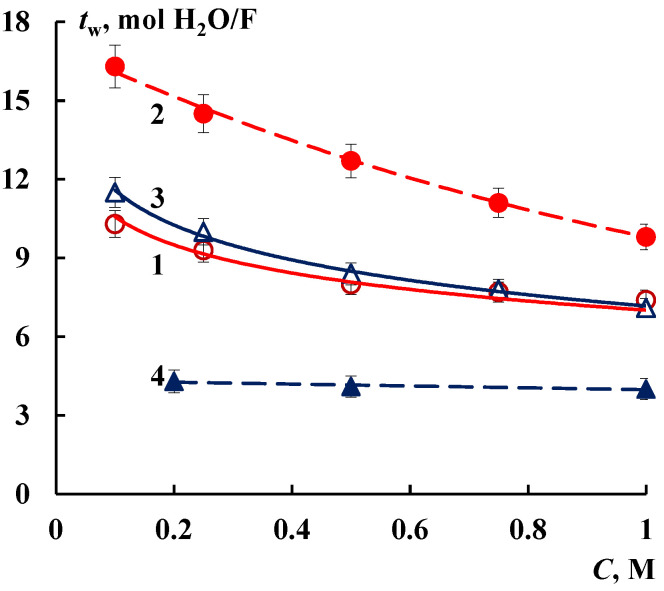
Concentration dependences of water transport numbers in NaCl solutions: 1—Nafion, 2—Nafion/SiO_2_, 3—MF-4SK, 4—MF-4SK/SiO_2_.

The changes in the structure of modified membranes influence on their electrotransport properties. The conductivity of Nafion/SiO_2_ membrane increases by 40% compared to the initial membrane (Figure 3). The formation of silica nanoparticles in the perfluorinated membrane leads to change in the transfer of not only ions but water as well. Figure 4 shows that the water transport number through Nafion/SiO_2_ membrane grows essentially in comparison with the initial membrane. The increase in the *t_w_* value is 20–50% depending on the NaCl solution concentration. The sample with such characteristics is promising for application in proton exchange membrane fuel cells (PEMFC) because of the so-called “water management” [42].

At the same time, the conductivity of MF-4SK/SiO_2_ membrane decreases by 5 times. It is accompanied by decrease in the water transport numbers by 2-3 times that achieve a minimum value about 4 mol H_2_O/F (Figure 4). It means that only water molecules of the first hydration shell migrate through the modified membrane [43,44]. It can be concluded that these materials with reduced water transport numbers and high selectivity are prospective for the application in separation processes. According to [17] the degree of concentration of NaCl solution in the electrodialyzer with MF-4SK/SiO_2_ membrane increases by 1.5 times. Thus, the intercalation of a dopant of the same type makes it possible to prepare membrane samples for various applications due to different modification conditions.

**Figure 5:**
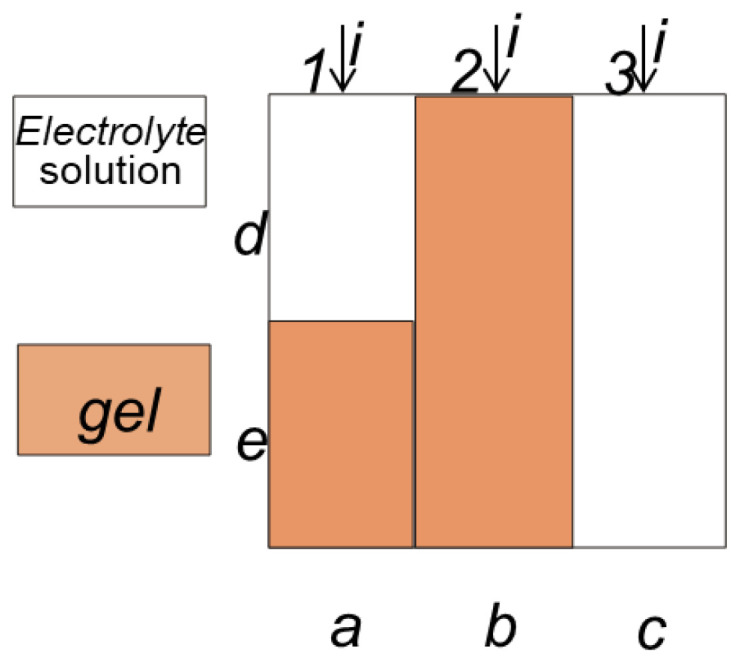
Scheme of current transfer in ion-exchange material within ETWM.

**Figure 6:**
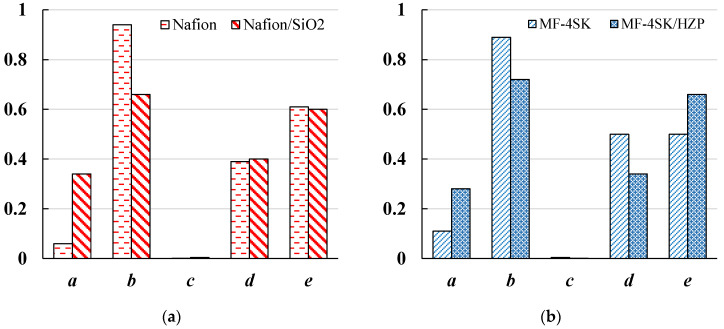
(**a**) A histogram of ETWM parameters for initial Nafion and (**b**) MF-4SK membranes and modi-fied Nafion/SiO_2_ and MF-4SK/HZP membranes.

Introduction of inorganic dopants into the membrane can change not only the conductivity value, but also the current flow mechanism because of structure reorganization. Therefore, the transport-structural parameters of extended three-wire model (ETWM) [45,46] were analyzed for Nafion/SiO_2_ and MF-4SK/HZP samples that are most promising for application in PEMFC. According to this model, the ion-exchange material consists of gel phase and inner equilibrium solution. The current is transferred by tree parallel channels: successively through gel phase and solution, only through gel phase and only through solution. The letters *a*, *b*, *c* in Figure 5 indicate fractions of current passing through mixed, gel and solution channels (*a* + *b* + *c* =1) correspondingly; *d* and *e* letters denote fractions of solution and gel in mixed channel *a* (*d* + *e* = 1). The transport-structural parameters of ETWM are calculated from concentration conductivity curves (Figure 3). ETWM parameter values are presented as histograms in the Figure 6.

The analysis of geometric ETWM parameters which characterize the distribution of conducting channels in the swollen polymer allows to obtain additional information about structural organization of the modified membranes. The conductivity in *c* channel is realized by the counter- and co-ions, so the value of *c* channel points out to the selectivity of the membrane. Analyzing the data presented in the Figure 6 we can conclude that the absence of the ***c*** channel indicates the preservation of the high selectivity of the modified membranes. For both modified membranes we observe an increase in the *a* value and a decrease in the *b* value. In addition, the ratio of gel (*e*) and solution (*d*) in the mixed channel after the modification by the HZP changes and the fraction of the gel phase increases.

Diffusion permeability of a membrane is significant transport characteristic. The diffusion permeability growth is usually accompanied by some decrease in membrane selectivity. Materials with reduced diffusion permeability are required for application in both electromembrane processes and electric current generation devices. The use of membranes with high diffusion permeability in redox flow batteries or PEMFC can be expected to reduce the device characteristics due to diffusion of neutral molecules or gases (crossover). Therefore, it is important to establish the influence of modification on the membrane diffusion permeability.

**Figure 7:**
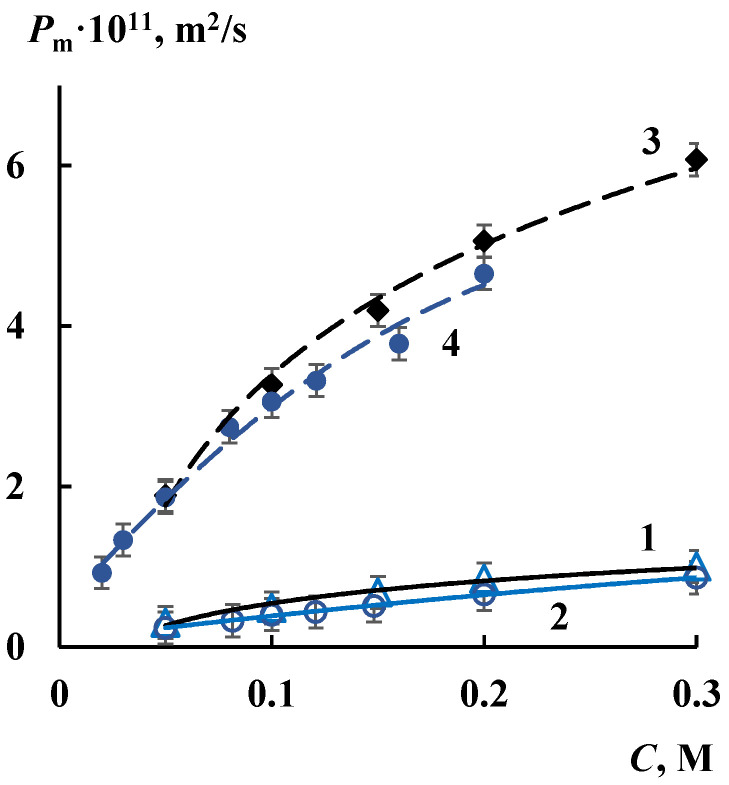
Concentration dependences of the integral diffusion permeability coefficient for MF-4SK (1, 2) and MF-4SK/HZP (3, 4) membranes in NaCl (1, 3) and HCl (2, 4) solutions.

Figure 7 shows the concentration dependences of the *P*_m_ value of the initial MF-4SK and the MF-4SK/HZP membranes in NaCl and HCl solutions. As can be seen, the intercalation of HZP into the perfluorinated membrane leads to an increase in the *P*_m_ value by approximately 6 times regardless of the electrolyte type. These results are consistent with an increase in the total volume of water in the hybrid MF-4SK/HZP membrane according to the integral porosimetric curves (Figure 2a) and the appearance of cavities with an effective size of more than 100 nm on the differential porosimetric curves (Figure 2b) in comparison with the initial membrane. 

A similar effect of increasing the diffusion permeability of the MF-4SK membrane after modification by silica under the same conditions as Nafion membrane was noted by the authors of [26]. Therefore, the *P*_m_ value of the MF-4SK membrane increases from 1.19 × 10^−11^ m^2^/s to 3.60 × 10^−11^ m^2^/s with the growth of NaCl solution concentration from 0.1 M to 1 M. The *P*_m_ value of the MF-4SK/SiO_2_ membrane changes from 3.20 × 10^−11^ m^2^/s to 7.70 × 10^−11^ m^2^/s at the same experimental conditions. Thus, the diffusion permeability of MF-4SK/SiO_2_ membrane is 2.4–3 times higher in comparison with initial membrane regardless of the concentration of the NaCl solution.

According to Figure 7 each membrane has the close values of diffusion characteristics in HCl and NaCl solutions because the diffusion process in ion-exchange materials is limited by co-ion transport, which is the same in this case. Such a significant increase in the *P*_m_ value is due to the treatment of the perfluorinated membrane by alcohol before modification. The diffusion permeability of the MF-4SK/HZP membrane decreases by 2–4 times in comparison with initial membrane [47] if such treatment is not carried out.

So, the increase in electroconductivity and the total water content is an advantage of modified membranes. However, a significant increase in diffusion permeability due to the pretreatment of the membranes with alcohol before modification is a negative factor. The increase in the diffusion permeability of electrolyte solutions through modified membranes indirectly indicates the possible increase in their gas permeability when operating in a fuel cell. The more the *P*_m_ value is, the greater hydrogen crossover will occur during the application in a PEMFC. Therefore, the value of membrane permeability in a salt or acid solution can be used for a qualitative estimation of the gas permeability and hydrogen crossover. A significant increase in the *P*_m_ value for Nafion/SiO_2_ and MF-4SK/HZP membranes limits their efficient application in fuel cells.

## 4. Conclusions

A correlation between changes in structural and electrotransport properties of membranes after modification by HZP and silica under different conditions was established. If the modification is accompanied by the growth of total water volume, it leads to increase in the conductivity and water transport numbers as well. Such effects are typical for perfluorinated membranes after modification by HZP and silica nanoparticles without heat treatment. The reduction of conductivity and electroosmotic permeability is usually associated with decrease in the membrane hydrate characteristics. Similar effects take place in the case of heat treatment of silica modified perfluorinated membranes. Such materials with reduced water transport numbers and high selectivity are prospective for the application in separation processes.

The concentration conductivity curves were used to characterize the distribution of conducting channels in the swollen polymer in the frame of the extended three-wire model. The analysis of model parameters proves the structure reorganization of hybrid membranes Nafion/SiO_2_ and MF-4SK/HZP compared with initial membranes.

Pretreatment of the membranes with alcohol before modifying by HZP and silica leads to the appearance of cavities with an effective radius more than 100 nm filled by free water with binding energy less than 10 J/mol. It is accompanied with an increase in the diffusion permeability of hybrid membranes by approximately 3–6 times in NaCl and HCl solutions. The value of membrane permeability to a salt or acid solution can be used for a qualitative estimation of the gas permeability and hydrogen crossover in electric current generation devices. A significant increase in the *P*_m_ value of Nafion/SiO_2_ and MF-4SK/HZP membranes limits their efficient application in both electrodialysis and fuel cells.

It was established that the hybrid membranes with different structure and transport properties can be prepared from perfluorinated membranes with similar characteristics and the same type of dopant under various conditions of modification such as pretreatment with alcohol before modification and heating after the synthesis of dopant nanoparticles in the membrane matrix.

## Figures and Tables

**Table 1:** Physicochemical characteristics of initial and modified perfluorinated membranes.

Membrane	*V*_0_, cm^3^/g_dry_	*Q*, mmol/g_dry_	*n*_m_, mol H_2_O/mol SO_3_^−^	$\frac{V_{\mathrm{micro}}}{V_{0}}$	$\frac{V_{\mathrm{macro}}}{V_{\mathrm{sw}.m}}$	*S*, m^2^/g	*L,* nm
**Nafion**	0.26	0.87	16.1	0.82	0.06	199	0.62
**Nafion/SiO_2_**	0.37	0.86	23.5	0.74	0.10	218	0.65
**MF-4SK**	0.23	0.80	16.2	0.85	0.05	194	0.63
**MF-4SK/SiO_2_**	0.18	0.72	13.7	0.88	0.03	194	0.67
**MF-4SK/** **HZF**	0.30	1.93	8.6	0.82	0.09	250	0.46

## Data Availability

Not applicable.

## Abbreviations

HZP	zirconium hydrophosphate
ETWM	extended three-wire model
PEMFC	proton exchange membrane fuel cells
